# Supplementary material for: Hypertension prevalence in patients attending tertiary pain management services, a registry-based Australian cohort study
Source: PLoS One. 2020 Jan 24;15(1):e0228173. doi: 10.1371/journal.pone.0228173 (PMC6980551; doi:10.1371/journal.pone.0228173)
Supplement: S1 Table — (DOCX) [file pone.0228173.s001.docx]

## **Supplementary materials**

S1 Table

*Comparison of adjusted ORs for the imputed dataset (N=43,789) and the cohort with complete data only (N=21,653)*

|  | Complete data only | Imputed dataset |
| --- | --- | --- |
|  | AOR (95% CI) | AOR (95% CI) |
| Sex ^c^ |  |  |
| Male | Reference | Reference |
| Female | **0.86 (0.80, 0.93)** | **0.88 (0.83, 0.93)** |
| Age ^d^ |  |  |
| 18 to 24 years | Reference | Reference |
| 25 to 34 years | **1.96 (1.19, 3.24)** | **1.87 (1.27, 2.75)** |
| 35 to 44 years | **3.57 (2.21, 5.79)** | **3.74 (2.58, 5.41)** |
| 45 to 54 years | **7.64 (4.74, 12.30)** | **7.40 (5.13, 10.67)** |
| 55 to 64 years | **15.03 (9.33, 24.20)** | **13.70 (9.50, 19.76)** |
| 65 to 74 years | **22.55 (13.94, 36.50)** | **20.29 (14.04, 29.34)** |
| 75 to 84 years | **34.01 (20.84, 55.50)** | **27.53 (18.96, 39.97)** |
| >= 85 years | **32.81 (19.09, 56.39)** | **30.65 (20.63, 45.55)** |
| Birth Region ^e^ |  |  |
| Oceania and Antarctica | Reference | Reference |
| Other | **1.13 (1.04, 1.22)** | **1.14 (1.08, 1.21)** |
| IRSAD Quintiles ^f^ |  |  |
| 5 (lowest disadvantage) | Reference | Reference |
| 4 | 1.08 (0.97, 1.21) | 1.05 (0.97, 1.14) |
| 3 | 1.09 (0.98, 1.21) | 1.08 (1.00, 1.16) |
| 2 | 1.05 (0.94, 1.17) | **1.10 (1.01, 1.19)** |
| 1 (highest disadvantage) | **1.14 (1.02, 1.28)** | **1.12 (1.03, 1.22)** |
| Body Mass Index ^g^ |  |  |
| Normal weight | Reference | Reference |
| Underweight | **0.71 (0.51, 0.99)** | 0.81 (0.63, 1.04) |
| Overweight | **1.74 (1.57, 1.93)** | **1.60 (1.47, 1.74)** |
| Obese, Class I | **2.55 (2.28, 2.84)** | **2.30 (2.09, 2.52)** |
| Obese, Class II | **3.30 (2.90, 3.76)** | **2.90 (2.62, 3.21)** |
| Obese, Class III | **4.74 (4.12, 5.44)** | **3.89 (3.47, 4.36)** |
| Comorbidities |  |  |
| Arthritis | **1.38 (1.28, 1.50)** | **1.50 (1.42, 1.59)** |
| Diabetes ^h^ | **2.37 (2.16, 2.61)** | **2.42 (2.26, 2.60)** |
| Anxiety ^i^ |  |  |
| Normal/mild | Reference | Reference |
| Moderate | 1.07 (0.96, 1.19) | 1.07 (1.00, 1.16) |
| Severe/Extremely severe | **1.29 (1.17, 1.43)** | **1.26 (1.17, 1.36)** |
| Depression ^j^ |  |  |
| Normal/mild | Reference | Reference |
| Moderate | 0.94 (0.84, 1.05) | **0.91 (0.84, 0.98)** |
| Severe/Extremely severe | **0.85 (0.76, 0.95)** | **0.87 (0.80, 0.95)** |
| Pain Source ^k^ |  |  |
| Post-injury | Reference |  |
| Post-surgery | **1.15 (1.02, 1.30)** | **1.10 (1.01, 1.20)** |
| Related to illness | 1.11 (0.99, 1.25) | 1.09 (1.00, 1.18) |
| No obvious cause | **1.12 (1.01, 1.24)** | 1.06 (0.99, 1.14) |
| Other cause | 1.07 (0.95, 1.19) | 1.08 (1.00, 1.17) |
| Pain Duration ^l^ |  |  |
| 3 to 12 months | Reference | Reference |
| 12 to 24 months | 1.03 (0.90, 1.19) | 1.02 (0.93, 1.13) |
| 2 to 5 years | 1.02 (0.90, 1.16) | 1.06 (0.97, 1.16) |
| > 5 years | 1.10 (0.98, 1.23) | 1.09 (1.00, 1.18) |
| Widespread Pain Index |  |  |
| < 3 sites | Reference | Reference |
| 3-6 sites | 1.02 (0.93, 1.12) | 1.00 (0.94, 1.06) |
| 7+ sites | 1.08 (0.98, 1.19) | 1.06 (0.99, 1.14) |
| Pain Severity ^m^ |  |  |
| Low | Reference | Reference |
| Moderate | **1.27 (1.09, 1.48)** | **1.15 (1.03, 1.29)** |
| High | **1.23 (1.04, 1.45)** | **1.17 (1.04, 1.31)** |
| Pain Interference ^n^ |  |  |
| Low | Reference | Reference |
| Moderate | 1.04 (0.89, 1.22) | 1.00 (0.89, 1.12) |
| High | 1.03 (0.87, 1.22) | 0.99 (0.88, 1.12) |
| Pain Self-Efficacy ^o^ |  |  |
| Low impairment | Reference | Reference |
| Mild impairment | 1.12 (0.95, 1.31) | 1.07 (0.96, 1.19) |
| Moderate impairment | 1.06 (0.92, 1.24) | 1.00 (0.90, 1.10) |
| Severe impairment | 1.04 (0.89, 1.20) | 1.02 (0.92, 1.13) |
| Pain Catastrophizing ^p^ |  |  |
| Clinically normal | Reference | Reference |
| High | 1.02 (0.92, 1.14) | 1.01 (0.93, 1.09) |
| Clinically elevated | 1.02 (0.91, 1.14) | 0.99 (0.91, 1.07) |
