# Supplementary material for: Hypertension prevalence in patients attending tertiary pain management services, a registry-based Australian cohort study
Source: PLoS One. 2020 Jan 24;15(1):e0228173. doi: 10.1371/journal.pone.0228173 (PMC6980551; doi:10.1371/journal.pone.0228173)
Supplement: S2 Table — (DOCX) [file pone.0228173.s002.docx]

## **Supplementary materials**

**S2 Table**

Levels of pain interference in patients with and without hypertension

|  | Hypertension | |  | No hypertension | |  |  |  |
| --- | --- | --- | --- | --- | --- | --- | --- | --- |
|  | *n* | *m (sd)* |  | *n* | *m (sd)* |  | *p* | *Hedges g (95% CI)* |
| General activity | 10,223 | 7.38 (2.25) |  | 32,443 | 7.29 (2.29) |  | 0.001 | -0.04 (-0.06, -0.02) |
| Mood | 10,195 | 6.98 (2.61) |  | 32,420 | 7.18 (2.51) |  | <0.001 | 0.08 (0.06, 0.10) |
| Walking | 10,232 | 7.09 (2.71) |  | 32,404 | 6.39 (2.96) |  | <0.001 | -0.24 (-0.26, -0.22) |
| Work | 10,122 | 7.59 (2.34) |  | 32,200 | 7.49 (2.39) |  | <0.001 | -0.04 (-0.06, -0.02) |
| Relationships | 10,102 | 5.90 (2.99) |  | 32,159 | 6.13 (2.94) |  | <0.001 | 0.08 (0.06, 0.10) |
| Sleep | 10,212 | 7.13 (2.70) |  | 32,377 | 7.28 (2.64) |  | <0.001 | 0.06 (0.03, 0.08) |
| Enjoyment of life | 10,131 | 7.55 (2.48) |  | 32,253 | 7.64 (2.46) |  | 0.002 | 0.04 (0.01, 0.06) |

Notes: Significance evaluated using independent samples t-test.
